# Annexin A2 overexpression associates with colorectal cancer invasiveness and TGF-ß induced epithelial mesenchymal transition via Src/ANXA2/STAT3

**DOI:** 10.1038/s41598-018-29703-0

**Published:** 2018-07-26

**Authors:** Murilo R. Rocha, Pedro Barcellos-de-Souza, Annie Cristhine M. Sousa-Squiavinato, Priscila V. Fernandes, Ivanir M. de Oliveira, Mariana Boroni, Jose A. Morgado-Diaz

**Affiliations:** 1 Cellular and Molecular Oncobiology Program, Instituto Nacional de Câncer, INCA, Rua André Cavalcanti, 37, 20231-050 Rio de Janeiro, Brazil; 2 Pathology Division - DIPAT, Instituto Nacional de Câncer, INCA, Av Cordeiro da Graça, 156, 20220-400 Rio de Janeiro, Brazil; 3 Bioinformatics Unit, Instituto Nacional de Câncer, INCA, Rua André Cavalcanti, 37, 20231-050 Rio de Janeiro, Brazil

## Abstract

Annexin A2 (ANXA2) is upregulated in several malignancies, including colorectal cancer (CRC). However, there is little knowledge on the molecular mechanisms involved to its upregulation. The aim of this study was to identify the mechanism through which ANXA2 overexpression leads to CRC progression and evaluate its potential prognostic value. We used human CRC samples to analyse the correlation between ANXA2 levels and tumour staging. ANXA2 expression was increased in CRC tissues compared to normal colon tissues. In addition, we observe increased ANXA2 levels in stage IV tumours and metastasis, when compared to stage I-III. Whereas E-cadherin, an epithelial marker, decreased in stage II-IV and increased in metastasis. We’ve also shown that TGF-β, a classic EMT inductor, caused upregulation of ANXA2, and internalization of both E-cadherin and ANXA2 in CRC cells. ANXA2 silencing hindered TGF-*β*-induced invasiveness, and inhibitors of the Src/ANXA2/STAT3 pathway reversed the EMT. *In silico* analysis confirmed overexpression of ANXA2 and association to the consensus moleculars subtypes (CMS) with the worst prognosis. Therefore, ANXA2 overexpression play a pivotal role in CRC invasiveness through Src/ANXA2/STAT3 pathway activation. The association of ANXA2 to distinct CMSs suggests the possible use of ANXA2 as a prognostic marker or directed target therapy.

## Introduction

CRC is the third most commonly diagnosed cancer and, in 2012, it was responsible for over 690 thousand deaths and accounted for 1.4 million new cases. Indeed, CRC’s heterogeneity has eluded researchers in the attempt to clearly characterize and define treatment strategies and markers that work for most of the patients. To tackle this problem, a consortium has analysed over 4,150 patients with this cancer and identified four consensus molecular subtypes (CMS) for CRC. For a review about the characteristic of these subtypes (See^1^). However, the increase in incidence rates among developing countries and the unexplained raise in number of cases among young adults from countries where incidence was decreasing^2^, calls for a better understanding of CRC progression and its diagnostic and prognostic markers.

Annexins are a family of calcium-dependent phospholipid-binding proteins involved in membrane trafficking and organization^3^. Annexin A2 (ANXA2), one of the twelve human annexins, has been described in endocytic and exocytic events^4^, and in actin cytoskeleton regulation^5^. Importantly, ANXA2 overexpression has been linked to a variety of tumours^6^, including colorectal cancer (CRC).

The EMT is a metastable cellular process, during which cells lose their apical-basal polarity, disassemble their cell-cell junctions and gain mesenchymal characteristics, and enhanced migratory and invasive capabilities^7^. These features make EMT-related proteins interesting in the attempt to prevent metastasis. In this context, ANXA2 has already been described as necessary for transforming growth factor beta (TGF-ß)- and epidermal Growth Factor (EGF)-induced EMT in pancreatic ductal carcinoma^8^ and breast cancer^9^. However, its role in CRC has not been described, yet.

In this study, we sought to confirm if ANXA2 is overexpressed in CRC human samples by analysing its expression in different stages of the disease, evaluate its prognostic value potential and explore the pathways through which ANXA2 overexpression leads to cancer progression. We found that ANXA2 is overexpressed in CRC samples, particularly in invasive tumours. Additionally, TGF-ß, a well-known inductor of EMT, led to ANXA2 overexpression, while inhibition of the Src/ANXA2/STAT3 pathway prevented the drastic phenotype changes and invasion induced by TGF-ß treatment. *In silico* analysis revealed ANXA2 association to specific CMS groups of CRC characterizing its role in CRC progression, distinct phenotypes, and an inverse relation with E-cadherin expression. Additionally, our *in vitro* results demonstrate the role of ANXA2 in the regulation of EMT and internalization of E-cadherin, through the Src/ANXA2/STAT3 axis. Finally, the association of ANXA2 levels to distinct CMSs suggests the possible use of ANXA2 as a prognostic marker or directed target therapy.

## Results

### ANXA2 is overexpressed in stage IV tumours and metastases

**Figure 1:**
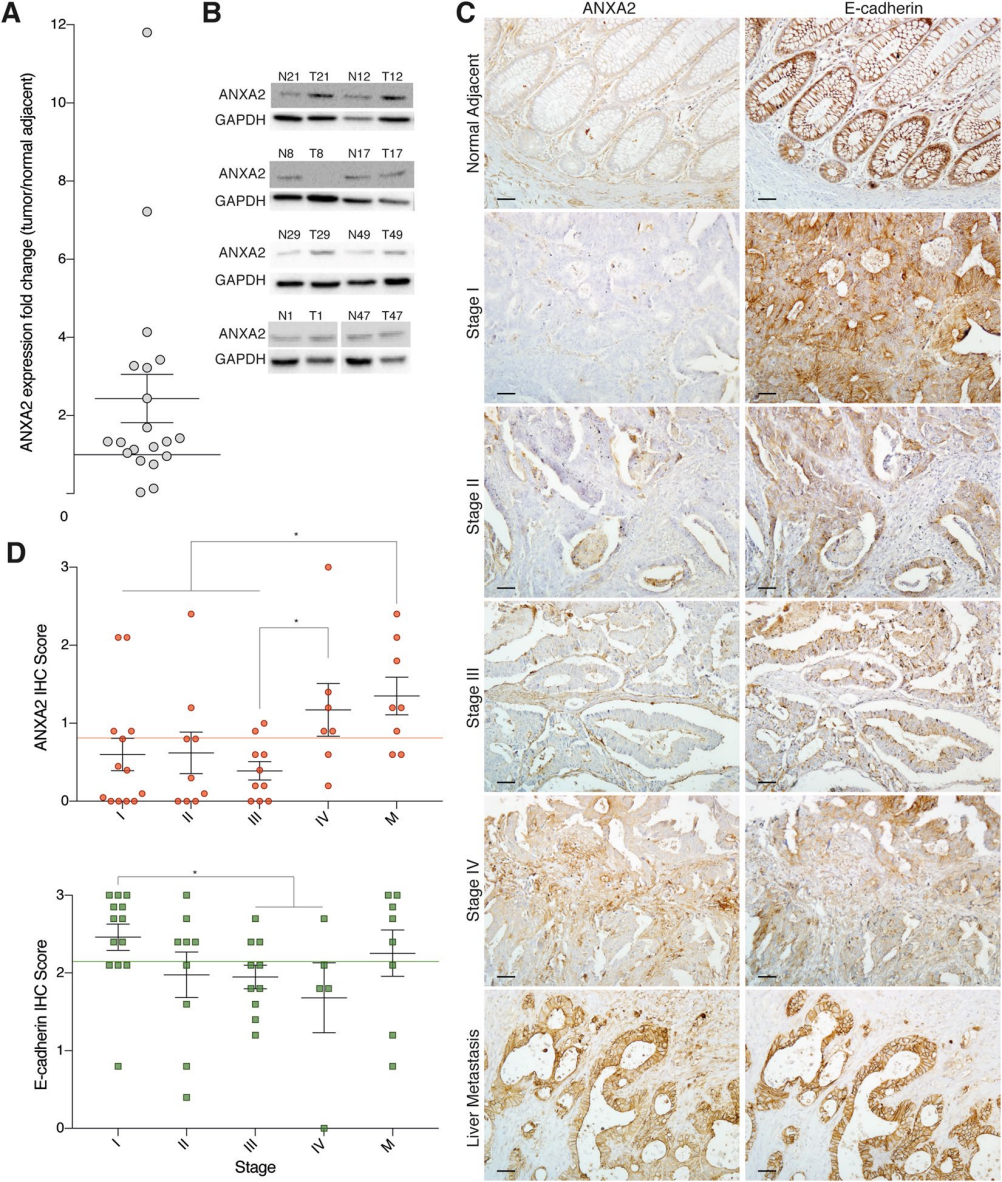
ANXA2 is overexpressed and E-cadherin is downregulated during CRC progression. **(A)** Graph of ANXA2 expression in total tumour lysates (n = 20) compared to normal adjacent paired sample. **(B)** Representative western blots from 8 (out of 20) patients with scores plotted in (**A**). **(C)** IHC images for ANXA2 and E-cadherin staining in different tumour stages (I-IV) and metastatic lesions. Scale bar = 50 μm. **(D)** Quantification of the IHC data. The IHC score was calculated as intensity x area of staining. The line represents the mean score. *p < 0.05 (paired t-student test).

Analysis of ANXA2 levels in 20 tumours and their adjacent areas revealed a mean ANXA2 overexpression in tumours of 2.43 times, in a range from 1,180% upregulation to 89% downregulation (Fig. 1A,B). Because of this heterogeneity, we selected 39 formalin fixed and paraffin embedded primary tumour samples and 8 secondary tumours (liver metastasis), and their normal adjacent counterparts, to evaluate ANXA2 expression. As observed in Fig. 1C, there is little to none ANXA2 staining in the initial stages (I, II and III) of CRC progression and a marked increase in ANXA2 levels in stage IV tumours and metastatic lesions. As E-cadherin downregulation is a hallmark of EMT; we evaluate EMT role in CRC progression by investigating E-cadherin levels in the same samples. As expected, E-cadherin staining intensity decreases in advanced tumour stages (III and IV), compared with healthy tissues, and regains its strength in metastatic lesions suggesting the occurrence of MET (Fig. 1C,D). ANXA2 mean immunohistochemistry (IHC) score (intensity x staining percentage) was used to define high- and low-ANXA2 tumours and assess the correlation of ANXA2 expression with tumour’s clinical features. ANXA2 overexpression correlated only with the presence of distant metastasis (p = 0.05, Supplementary Table 1). Interestingly, ANXA2 exhibited two distinct staining patterns in secondary tumours. ANXA2 staining was similar to that of E-cadherin (intense in cellular membranes) in small metastases. However, while E-cadherin stained the entire lesion regardless of its size, ANXA2 was present only at the edges of large metastatic lesions (Supplementary Fig. 1).

### TGF-ß-induced EMT leads to ANXA2 overexpression and subcellular redistribution

**Figure 2:**
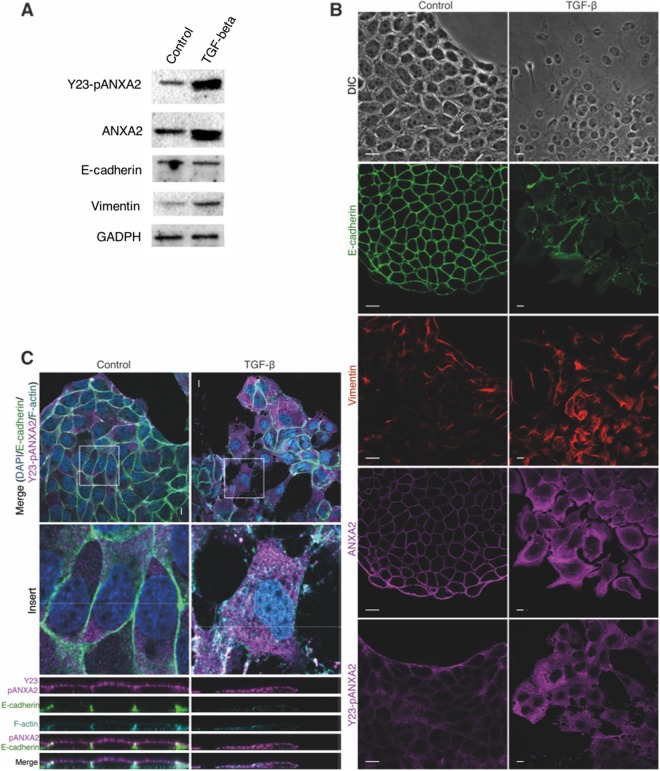
TFG-ß treatment induces EMT and overexpression and re-distribution of ANXA2. **(A)** Western blot relative to ANXA2 (total and phosphorylated), and epithelial (E-cadherin) and mesenchymal (vimentin) markers after 48 h of TGF-ß treatment. **(B)** Immunofluorescence showing re-distribution of ANXA2, E-cadherin and vimentin along with the DIC representative images. **(C)** Confocal images and z-axis analysis of control and TGF-ß-treated cells in quadruple immunofluorescence (DAPI/E-cadherin/Y23-pANXA2/F-actin). Scale bar = 10 μm.

In order to check if ANXA2 overexpression, followed by E-cadherin downregulation, is essential to CRC cells invasion, HT-29 cells were treated with 10 ng/ml of TGF-ß for 48 h. Western blot analysis revealed that both ANXA2 and its phosphorylated form (Y23-pANXA2) are upregulated upon TGF-ß treatment. E-cadherin (epithelial marker) seemed unaltered, while the expression of vimentin (mesenchymal marker) increased (Fig. 2A). We then performed immunofluorescence assays to investigate whether the subcellular localization of ANXA2 changes after TGF-ß treatment (Fig. 2B). Differential interference contrast (DIC) images shows the loss of cell-cell contacts and an elongated phenotype in TGF-ß-treated cells. E-cadherin, localized at the cellular junctions in untreated cells (control) and is internalized but not degraded after 48 h of treatment. This result explains why there was no significative change in E-cadherin expression in the western blot analysis. Vimentin, although present in HT-29 control cells, exhibited a more intense staining in the EMT-induced group. Total ANXA2 and Y23-pANXA2, localized at the cellular junctions in control cells, were overexpressed and redistributed to the cytoplasm in TGF-ß-treated cells. It is well known that EMT causes the loss of cellular junctions and apical-basal polarity. Therefore, we further investigated these changes through confocal microscopy. Specifically, we stained HT-29 cells for Y23-pANXA2, E-cadherin and F-actin (phalloidin staining) (Fig. 2C). E-cadherin, localized at the cell-cell contacts in control cells, localizes to the cytoplasm upon TGF-ß treatment. Y23-pANXA2 mostly localizes at the apical membrane in control cells, but distributes to the whole cell after TGF-ß treatment. Finally, F-actin staining highlighted the change in shape (from columnar to fusiform) of the TGF-ß-treated cells (Fig. 2C).

### Inhibition of the Src/ANXA2/STAT3 pathway prevents EMT induction and TGF-ß-induced cell invasion

**Figure 3:**
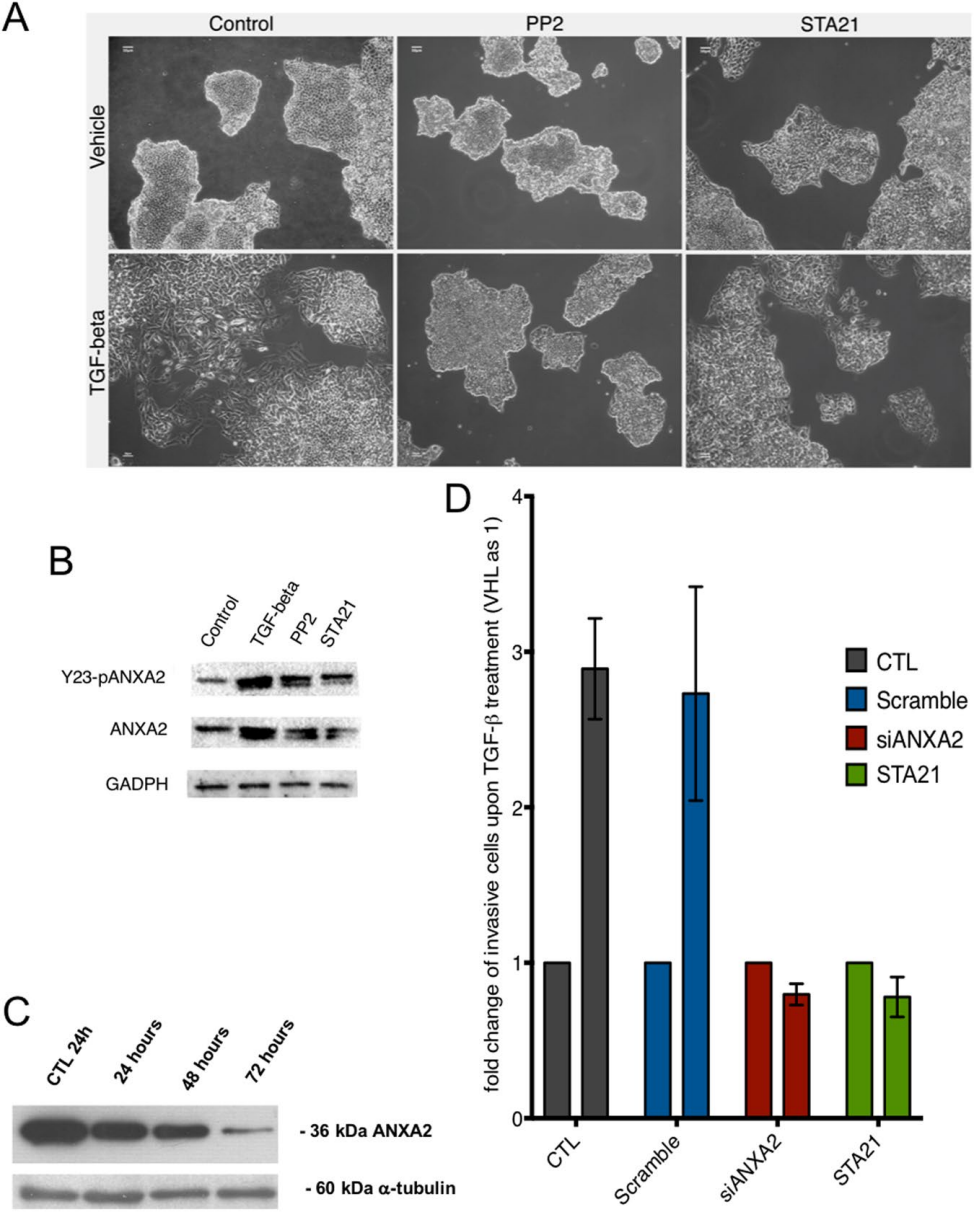
The Src/ANXA2/STAT3 pathway regulates TGFB-induced EMT and cellular invasion. **(A)** DIC images representative of the cellular phenotype after TGF-ß treatment with or without pre-treatment with Src (PP2) and STAT3 (STA21) inhibitors. **(B)** Western blot for ANXA2 and Y23-pANXA2 after treatment of the cells with TGF-ß, PP2 or STA21, as indicated. (**C**) Western blot of ANXA2 expression after ANXA2 siRNA transfection. (**D**) Graph of Matrigel^®^ invasion assays: the number of invasive cells was normalized to the control in each group.

Previous studies have described the importance of ANXA2 Y23 phosphorylation for TGF-ß-induced EMT in pancreatic ductal carcinoma^8^. Additionally, the Src/ANXA2/STAT3 pathway has been implicated in breast cancer invasion and metastasis formation^9^. Therefore, we investigated whether the Src/ANXA2/STAT3 plays a role in EMT in our system. We pre-treated the cells with a Src inhibitor (PP2) or a STAT3 selective inhibitor (STA21) 1 h prior to TGF-ß treatment. PP2 completely inhibited EMT, with HT-29 colonies showing a condensed epithelial morphology (Fig. 3A). On the other hand, STAT3 inhibition partially prevented cellular alterations due to the EMT. Next, we investigated whether Src or STAT3 inhibition affects ANXA2 levels. Western blot assays revealed that PP2 and STA21 treatment partially prevented the ANXA2 and Y23-ANXA2 overexpression induced by TGF-ß (Fig. 3B). Then, we knocked down ANXA2 expression through RNA interference and analysed how ANXA2 levels interfered with TGF-ß effects. In a preliminary experiment, we observed the peak in silencing 72 h after transfection of the siRNAs (Fig. 3C). Cell invasion experiments revealed that ANXA2 silencing, similarly to STAT3 inhibition, could prevent cell invasion induced by TGF-ß (Fig. 3D). ANXA2 silencing also hindered cellular proliferation and diminished HT-29 cells’ wound closure capabilities (Supplementary Fig. S2). Both actions were independent from the anti-proliferative and anti-migration effect of TGF-ß.

### Bioinformatics analysis of *ANXA2* and *CDH1* expression in different tumour stages and CRC subtypes

**Figure 4:**
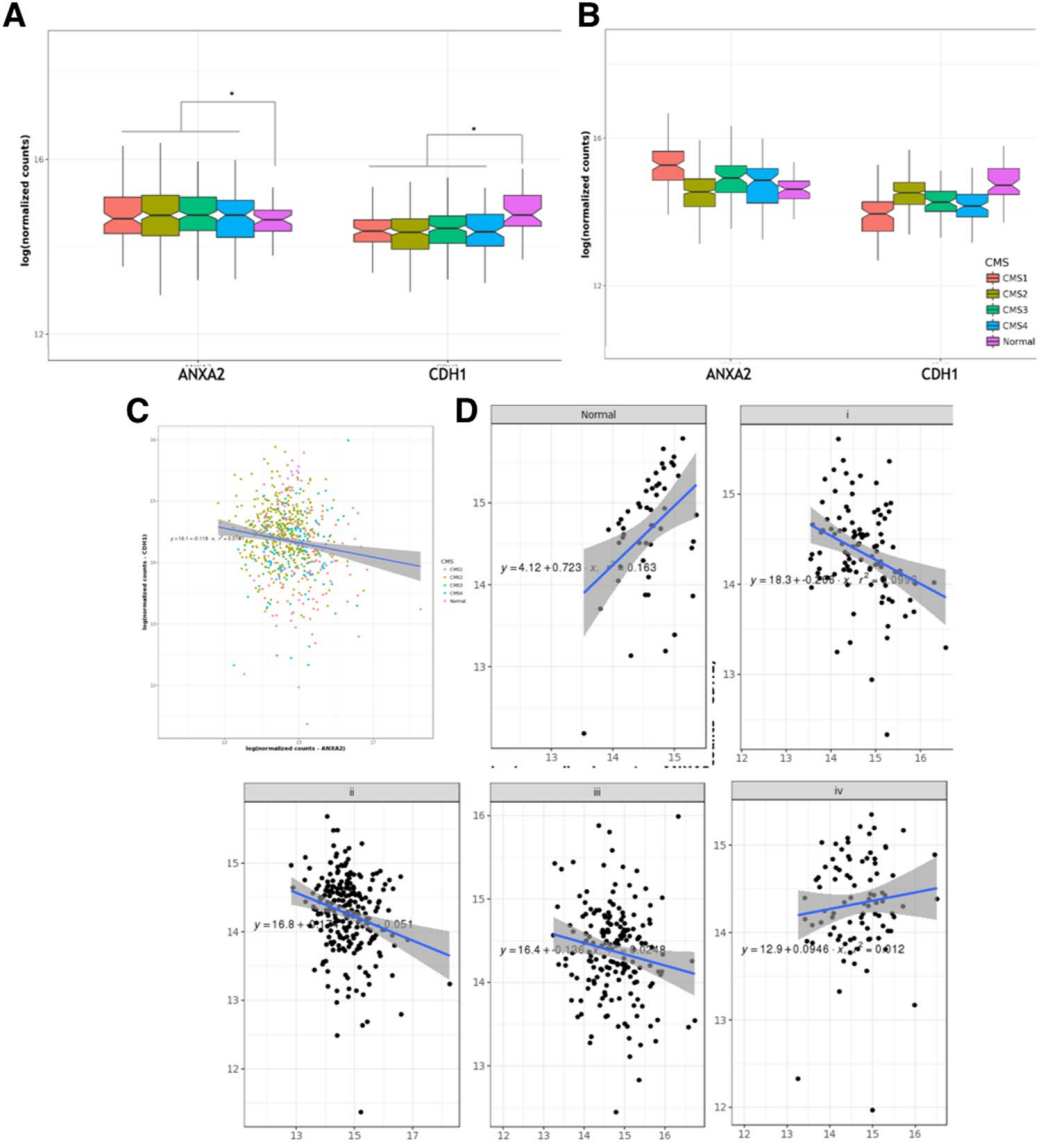
*In silico* analysis of *ANXA2* and *CDH1* expression in colon and rectal adenocarcinomas using TCGA-COAD and TCGA-READ data banks. (**A**) Expression of *ANXA2* and *CDH1* according to the CRC stage, compared to the control. Groups are coded by the following colors: Stage 1 – Red/Stage 2 – Cargo Green/Stage 3 – Green/Stage 4 – Blue/Control group – Purple. (**B**) Patients were clustered according to the CMS classification and *ANXA2* and *CDH1* expression in each subtype was plotted. All groups differ from each other (p < 0.005). (**C**) Correlation between *ANXA2* and *CDH1* in all stages. (**D**) Correlation between *ANXA2* and *CDH1* per tumour stage. *p < 0.001 (Wilcoxon rank sum test and Bonferroni post-hoc).

To corroborate the findings of our *ex vivo* and *in vitro* assays, we performed an *in-silico* analysis on RNAseq data from 633 patients with colon or rectal adenocarcinoma (TCGA-COAD and TCGA-READ databases). The expression of *ANXA2* and *CDH1* was evaluated in cancer (at different stages) and healthy datasets. *ANXA2* was upregulated in all cancer stages (I–IV) when compared to normal controls. Contrarily, *CDH1* expression was downregulated in all stages (Fig. 4A). Taking into account the heterogeneity of CRC, we used an algorithm for the classification of the patients into the CMS and analysed how *ANXA2* and *CDH1* expression clustered inside these subtypes. All CMS were statically different for *ANXA2* and *CDH1* expression, with an interesting inverse relation between the two molecules. The groups with higher *ANXA2* displayed lower *CDH1* expression. *ANXA2* showed higher expression in CMS1 (MSI/immune), followed by CMS3 (metabolic), CMS4 (mesenchymal), CMS1 (canonical) and control. CDH1 expression was lower in CMS1, followed by CMS4, CMS3, CMS2 and control (Fig. 4B). Given the inverse relation between *ANXA2* and *CDH1*, we checked if there was indeed a correlation between the two. However, we did not find any significant correlation either in the global or in the stage specific data (Fig. 4C).

### ANXA2 and E-cadherin co-localize at cellular junctions and are internalized together upon TGF-ß treatment

**Figure 5:**
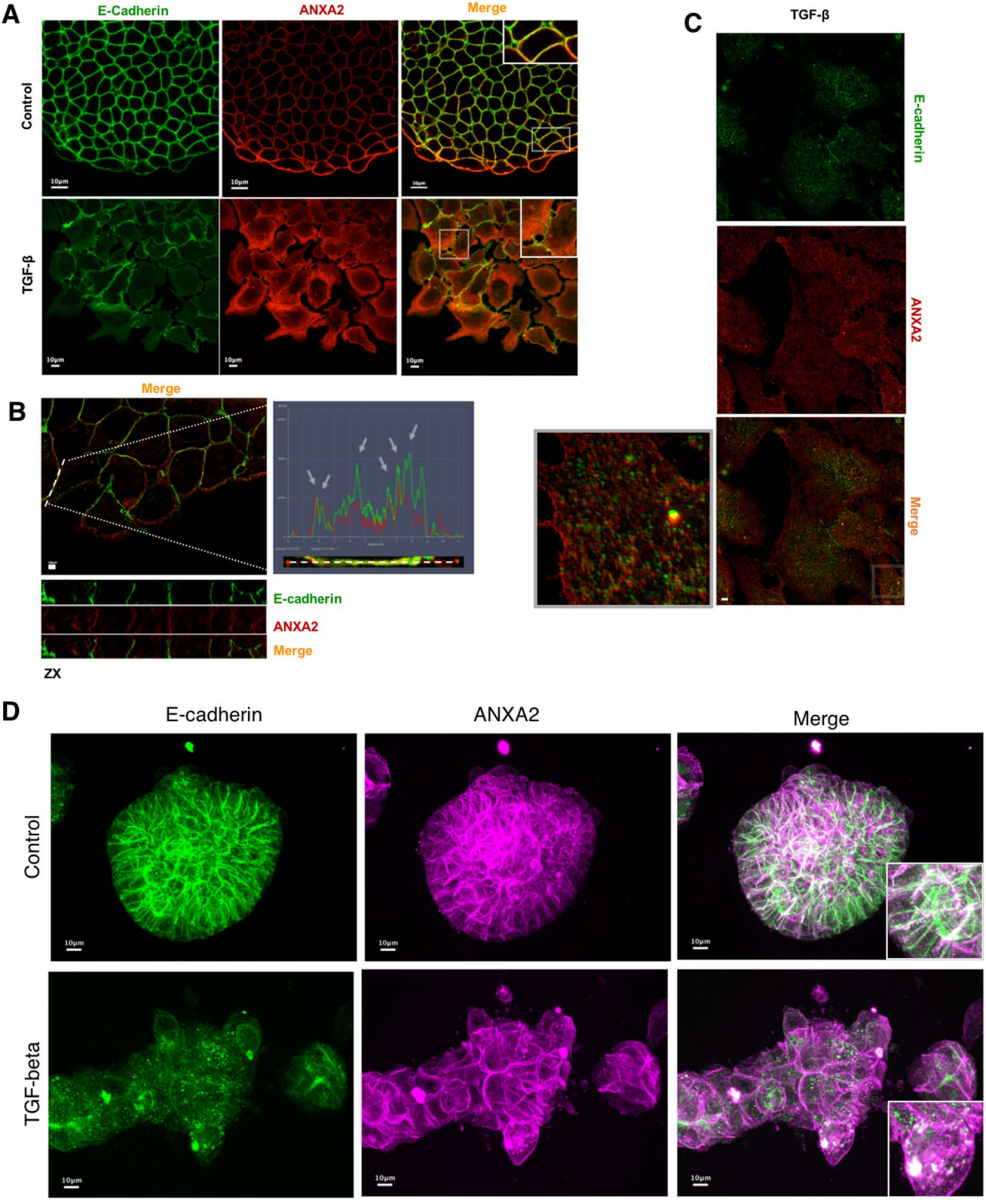
ANXA2 co-localizes with E-cadherin and participates in its internalization upon TGF-ß treatment. (**A)** Confocal images relative to ANXA2 and E-cadherin localization upon TGF-ß treatment. **(B)** Structured illumination microscopy analysis of ANXA2 and E-cadherin in the control group and the group treated with TGF-ß **(C)**. In this later image, it is possible to observe E-cadherin and ANXA2 co-localization in dotted cytoplasmic structures. **(D)** Maximum projection of immunofluorescence of spheroids exhibiting similar features (ANXA2 and E-cadherin co-localization) to the monolayer cultures treated or without treated with TGF-ß. Scale bars = 10 μm.

In order to better comprehend ANXA2 and E-cadherin relation during EMT development, we performed double immunofluorescence staining assays. Confocal microscopy showed that control HT-29 cells exhibited co-localization of the two proteins at the cellular contacts. However, due to the overexpression of ANXA2 in TGF-ß-treated cells, we could not discern if, in these cells, the two proteins co-localized or the co-localization observed was a technical artefact (Fig. 5A). To solve this issue, we employed structured illumination microscopy, a technique that allows superior resolution when compared to conventional confocal microscopy. Fluorescence intensity profile analysis of one cell contact region indicated the interaction between E-cadherin and ANXA2 in the cellular junctions of control cells (Fig. 5B). TGF-ß-treated cells displayed both proteins internalized with the highest intensity regions co-localizing (Fig. 5C). To better analyse our findings, obtained in 2D system, we developed a 3D model of early EMT (Supplementary Fig. S3): DIC images of the spheroids generated, confirmed the change in their structure upon TGF-ß treatment. Notably, confocal microscopy indicated that, in the spheroids, ANXA2 and E-cadherin co-localized at the cell-cell junctions. In TGF-ß-treated spheroids, ANXA2 and E-cadherin co-localized in the cytoplasm, possibly in vesicles present close to the cell-cell junctions (Fig. 5D).

## Discussion

Early studies have described ANXA2 overexpression in an increasing amount of tumour types including CRC. However, no mechanistic studies related with this overexpression or even describing their prognostic value in this last cancer type, has been reported. In this study, we found that *ANXA2* mRNA is upregulated at all stages of CRC and ANXA2 protein levels (evaluated by IHC staining and immunoblotting) associate with high probability of invasion and distant metastasis. Despite its mRNA overexpression in all tumoral stages according to the TCGA data, the distinct protein levels between TNM stages found in our samples might be explained by two components. First, changes in mRNA stability may induce a higher protein level with similar mRNA expression. It’s been shown that upon TGF-ß treatment, ANXA2 mRNA expression reaches a 16 h peak returning to its control amount in 24 h. The same does not happen with protein levels, that reach a peak at 24 h and maintain its overexpression until 72 h^9^. Second, post-translational modifications can alter ANXA2 protein stability, function and degradation such as its ubiquitination^10^. The evidence of ANXA2 overexpression in metastatic lesions, after cells would have undergone MET (and thus re-express E-cadherin), is another indication that EMT is a metastable program with several intermediate steps^7^ and we reported by the first time in CRC in the present study. The fact that larger metastatic lesions expressed ANXA2 only on the edges could be explained by the role of this protein in interacting with stromal cells to promote tumour growth, as described in metastatic lesions from pancreatic carcinoma^11^. Alternatively, ANXA2 might be cleaved in the necrotic or apoptotic cells in the centre of the metastatic lesion. This possibility is, however, less likely than the previous one. The staining pattern we observed has already been described in subcutaneous tumours, where ANXA2 is only expressed in the proliferating edges of the tumour^12^. The correlation between ANXA2 levels and CRC stages suggests that ANXA2 is important for EMT.

TGF-ß treatment in HT-29 cells also induced ANXA2 overexpression. This upregulation was later shown to be a consequence of the TGF-ß activation, because Src and STAT3 inhibitors could not fully reverse this effect. ANXA2 role in invasion has already been described through distinct mechanisms: extracellular matrix remodelling^13^, EGF pathway activation^14^ and, as in our system, STAT3 activation^15^. ANXA2 differential expression in CRC CMS suggests that this molecule plays an additional role besides regulating the EMT. Highly overexpressed in the CMS1 immune/microsatellite instability phenotype, ANXA2 could be responsible for STAT3 activation through multiple growth factors (since the Janus kinase (JAK)/STAT signalling is a characteristic of this subtype). Considering the TCGA data come from whole tissue lysates, ANXA2 might actually be overexpressed only in some cell types, like the immune infiltrate. It has already been shown that ANXA2 can activate STAT3 in macrophages and promote tumour formation^16^. Analysis of survival after relapse shows that CMS1 (MSI Immune) have the worst survival between the subtypes; which could be attributed to tumors aggressiveness and distant metastasis occurrence. ANXA2 overexpression in a subtype different from the CMS4 (with closer association to the EMT phenotype) can be explained by the fact that there is an overlap in CMS classification, where a subset of patients possesses markers of two subtypes. CMS4 (Mesenchymal) and CMS1 (MSI Immune) have such an interface^17^. Also overexpressed in CMS3 (metabolic subtype), ANXA2 could be upregulated as a consequence of K-Ras activation. In this regard, it is worth noticing that microRNA-206 (miR-206) downregulates both ANXA2 and K-Ras, and is under expressed in most CRC tumours^18^.

**Figure 6:**
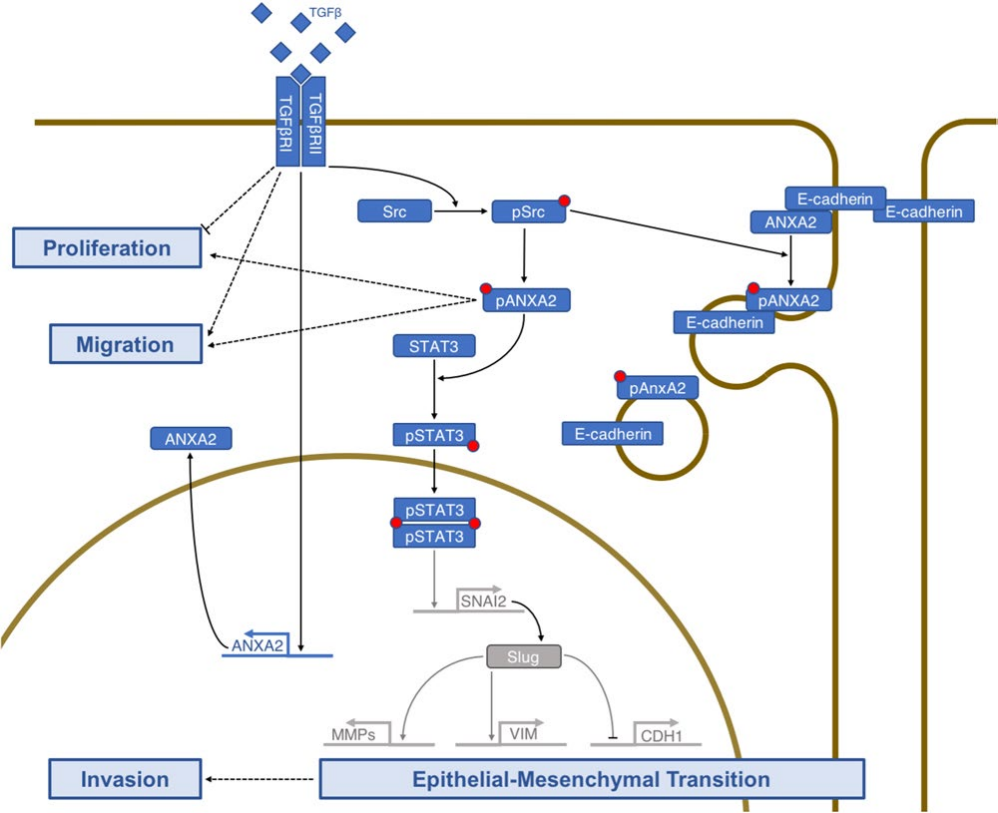
Model proposed of the EMT-related pathways regulated by ANXA2 after TGF-ß activation.

Regardless of which CMS was analysed, E-cadherin was downregulated when ANXA2 was overexpressed. However, there was no significant inverse correlation in an overall case assessment. This might be explained because of HT-29 cells characteristic of internalizing but not completely degrading E-cadherin. Hypothesis supported by findings of ANXA2 and E-cadherin co-localization in vesicles: ANXA2 might act on the removal of this junctional protein from cell-cell contacts. Previous studies indicate ANXA2 role in adherent junction dynamics^19^ and in maintaining the epithelial barrier integrity^20^. We propose that TGF-ß receptor activation leads to Src-mediated ANXA2 phosphorylation and ANXA2 overexpression. ANXA2, in turn, promotes E-cadherin internalization and STAT3 phosphorylation. STAT3 translocate to the nucleus and might induce SNAI2 (Slug) expression, which will downregulate *CDH1* transcription and upregulate vimentin and metalloproteinases expression (Fig. 6).

In conclusion, in this study, we highlight the importance of ANXA2 in CRC progression and its role in invasion via Src/ANXA2/STAT3 activation. Additionally, we demonstrate the correlation between ANXA2 expression and specific CMS of CRC and propose a new role for ANXA2 in tumorigenesis through the internalization of junctional proteins, specifically of E-cadherin, which is critical to maintain cell-cell contacts (Fig. 6). Being critical to tumour invasion pathways, ANXA2 association with distinct CRC subtypes places it as an important player in CRC progression, a useful tool for screening patients with CRC and a possible target for new drugs aimed to interfere with the EMT.

## Methods

### Reagents and antibodies

The mouse monoclonal anti-AnxA2 and anti-tubulin antibodies were purchased from Invitrogen Inc. (Carlsbad, CA, USA). The mouse monoclonal anti-AnxA2 phosphorylated on tyrosine 23 (Y23-pANXA2) antibody was purchased from R&D Systems (Minneapolis, MN, USA). The mouse monoclonal anti-E-cadherin antibody (clone 36) was purchased from BD Biosciences (San Diego, CA, USA). The mouse anti-glyceraldeyde 3-phosphate dehydrogenase (GAPDH) antibody was purchased from Santa Cruz (Dallas, TX, USA). Alexa Fluor^®^ 488 IgG-goat-anti-mouse and anti-rabbit and Alexa Fluor^®^ 546 (falsely coloured magenta to avoid colour blind misdirection) IgG-goat-anti-mouse antibodies were obtained from Molecular Probes (Eugene, OR, USA). 4-dicloridrato de 6-diamidine-2-fenilindole (DAPI) was purchased from Sigma-Aldrich (St. Louis, NO, USA). STAT3 selective inhibitor (STA-21) and Src inhibitor (PP2) were bought from Santa Cruz and reconstituted in dimethyl sulfoxide (DMSO) at stock concentration of 10 mM, stored at −20 °C and used at a final concentration of 10 μM. TGF-ß was purchased from Gibco (Chagrin Falls, OH, USA), reconstituted 0.1% BSA in filtered injection water and used at a final concentration of 10 ng/mL.

### Cell culture

The HT-29 cell line (HTB-38), was obtained from American Type Culture Collection (ATCC, Manassas, VA, USA) and maintained in the recommend culture conditions. For the experiments, cells were cultured in 6- or 12-well plates, flasks or polycarbonate filters transwell, with pores of 8 μm (Corning Inc., Corning, NY, USA).

Three-dimensional culture was performed covering coverslips with 50 μL of Matrigel^®^ (1:1) and placing them, individually, in a 12-well plate. Coverslips were left at 37 °C for complete polymerization. After 24 h, HT-29 cells were counted and 200 cells were resuspended in 200 μL of 50% Matrigel^®^ in Dulbecco’s modified Eagle’s medium (DMEM) and seeded in each well. Cells were cultured for three days prior to TGF-ß treatment and then maintained for three more days in the presence of TGF-ß.

### TGF-ß treatment and pharmacological inhibition

TGF-ß (10 ng/mL) was added to the cells after 12 h of culture in DMEM plus 1% foetal bovine serum (FBS). PP2 and STA-21 were added one hour prior to TGF-ß treatment.

### Tumour samples

Fresh samples of tumoral tissues and normal (healthy) adjacent tissue (at least 5 cm away from the lesion) from the primary tumour site of patients with CRC, were obtained from the Hospital do Câncer I – Instituto Nacional de Câncer (INCA) surgical centre (Rio de Janeiro, Brazil). The classification of the samples used in this study followed the guidelines from the *American Joint Committee on Cancer* (Edge *el al*., 2010). This study was approved by the Instituto Nacional de Cancer Ethics Research Committee (CEP-INCA) with the registry 84/04, updated on 17/09/2015, following all relevant guidelines and regulations. Informed consent was obtained from all subjects.

### Cell extraction and western blot

Protein extraction and western blot were performed as previously described^21^.

### Immunohistochemistry (IHC) and pathological analysis

Paraffin blocks stored in Pathology Division of INCA from 47 patients (39 with primary tumors and 8 with metastasis) that underwent colon resection surgery were selected. Staging of the tumor samples used in the study was performed according to the guidelines contained in the 7^th^ edition of the Cancer Staging Manual edited by the American Joint Commitee on Cancer (Edge *et al*., 2010). The staging of tumor samples was performed by Pathologists of INCA.

The IHC technique using sections of the selected blocks was performed on two consecutive days. The pre-treated commercial plates (immunoSlide-Easy Path) containing 3 micron slices were immersed in 3 × 5-minute baths followed by fast baths in 100%, 90%, 80% and 70% alcohol. The excess alcohol was withdrawn under running water for 3 minutes. Antigenic recovery was in Trilogy Buffer (Cell Marque), at 98 °C, using the steam process, for 30 min. Peroxidase blockade and protein blockade were done using the NovoLink Max Polymer Detection kit (Leica Microsystems) for 5 min each. Incubation with the mouse anti-E-cadherin primary monoclonal antibody at 1: 1200 dilution and anti-Annexin A2 monoclonal mouse primary antibody at 1:50 dilution was performed overnight at 4 °C.

On the second day, the slides were incubated with the post-primary antibody and the polymer (Novolink), both for 30 min. For the development of the reaction, the DAB chromogen was used for 3 min. The counter staining was done with hematoxylin for 30 seconds. After removal of excess hematoxylin in running water, the slides were immersed in 70%, 80%, 90%, 100% and xylol baths. After assembly, slides were analyzed under an optical microscope.

The slides were then sent to a pathologist (unfamiliar with the experimental groups) for evaluation of intensity (+ to +++) and percentage (0 to 100%) staining for each antibody on each slide. The results were then grouped and, for quantitative purposes, we defined a marking score. The score consisted on the multiplication of staining intensity (1 to 3 crosses) and percentage of the slide stained (0,00 to 1,00). For example, one slide where the pathologist score +++ crosses for intensity in 70% of the slide would have a score of 2, 1 (3 × 0,7 = 2, 1). The mean score among all patients was used for separation between high and low expression.

### Immunofluorescence

Cells were seeded on coverslips until reaching sub-confluence. The medium was then changed to DMEM plus 1% FBS for 12 h. Afterwards, the cells were treated with TGF-ß for 48 h, and fixed to investigate the localization of epithelial and mesenchymal markers. Immunofluorescence, microscopy and image acquisition protocols have been previously described^22^. Super-resolution images were taken using a LSM 710 microscope (Carl Zeiss, Germany) equipped with a PCO Edge sCMOS camera (PCO AG, Germany) using a Plan-Apochromat 63 Å~/1.4 Oil DIC M27 lens and 488- and 561-nm laser lines. Image acquisition, reconstruction, and alignment for structured illumination microscopy were performed using the Zeiss ZEN 2012 SP1 software (black edition, version 8.1.5.484). Contrast and colors were adjusted using ICY bioimage analysis software. (Institut Pasteur, Paris – France).

### Invasion assay

Cellular invasion was evaluated using transwell membranes (with pores of 8 μm) covered with Matrigel^®^ (1:10). HT-29 cells (2.5 × 10^4^) were seeded on the upper chamber of transwells pre-coated (24 h before) with Matrigel^®^ in DMEM plus FBS 1%, with or without TGF-ß; DMEM plus 10% FBS was placed in the inferior chamber, as a chemoattractant. After 48 h at 37 °C, the invading cells were fixed with methanol and stained with DAPI (1:1,000) for one minute and photographed in an inverted microscope (Zeiss Observer Z.1) coupled to the image processing software AxionVision Release 4.8.2 (Zeiss).

### Cell proliferation

Viable cell relative number was determined by crystal violet assay. After 24 h of silencing methodology, si-ANXA2 HT-29 cells (3 × 10^3^) were plated in 96-well plate for 4 hours. Cells were incubated in DMEM plus 1% or 10% FBS and treated with TGF-b for 24 or 48 hours. Culture media were removed, cells were PBS-washed, fixed with absolute ethanol for 10 min and crystal violet-stained (0,05% in ethanol 20% solution) at room temperature. After H_2_0-washing and air-drying, the dye was solubilized with methanol and optical densitometry (O.D) was assessed in a spectrophotometer (Spectra Max 190, Molecular Devices, Sunnyvale, CA, USA) at 595 nm. O.D. was evaluate using Soft Max pro 4.3 LS software.

### Wound Healing cell migration assay

HT-29 cells were seeded at 4 × 10^4^ cells/cm^2^. After reach confluency, cells were incubated with DMEM plus 1% FBS and a scratch was made by a pipette tip in cell monolayers. For each treatment, three scratches were made and three fields for each scratch were selected and further examined. After PBS washes, cultured media containing or not TGF-ß was added to the cells, and they were incubated at 37 °C. Images were captured at the beginning and after 24 h of incubation by inverted microscope (Zeiss Observer.Z1) coupled with an image processing software, AxioVision Release 4.8.2 (Zeiss). Clear areas were measured and compared using Icy Bioimage software.

### RNA silencing

The protocol used for the knockdown through small interfering RNA (siRNA) has been previously described in^22^. siRNA for AnxA2 (Annexin II siRNA (m), Cat. Sc-29683) and Control siRNA (Control siRNA-A, Cat. Sc-37007) were obtained from Santa Cruz.

### Gene expression analysis

For *ANXA2* and cadherin 1 (*CDH1*) expression analysis, we used RNAseq data from the Cancer Genome Atlas Colon Adenocarcinoma (TCGA-COAD) and Cancer Genome Atlas Rectal Adenocarcinoma (TCGA-READ) data banks. Tumour samples (633) and 51 normal colon and rectal samples were analysed. To evaluate *ANXA2* and *CDH1* expression in each stage and CMS we used a classifying algorithm, that allows the classification of each patient into CMS (https://github.com/Sage-Bionetworks/crcsc).

### Statistical analysis

All quantitative data are presented as the mean ± standard error (SEM) of at least three independent experiments. Statistical analysis and graphs were made using GraphPad^TM^Prism, 7.0 (GraphPad^TM^ Software, San Diego, CA, EUA). Variance analysis (ANOVA) of one or two ways was used with the Bonferroni post-test. TCGA data were analysed with the Wilcoxon rank sum, and Bonferroni post-hoc. Difference was considered significant when p < 0.05. All methods and experimental protocols were carried out in accordance with the relevant guidelines and regulations, and approved by the Instituto Nacional de Cancer (INCA) Ethics Research Committee (registry 84/04, updated on 17/09/2015).

### Data availability

The datasets generated during and/or analysed during the current study are available from the corresponding author on reasonable request.

## Electronic supplementary material

Supplementary Images

Supplementary Table I. Association of ANXA2 expression and clinicopathological features

Supplementary Table II. Reagents and Antibodies
