# Supplementary material for: Annexin A2 overexpression associates with colorectal cancer invasiveness and TGF-ß induced epithelial mesenchymal transition via Src/ANXA2/STAT3
Source: Sci Rep. 2018 Jul 26;8:11285. doi: 10.1038/s41598-018-29703-0 (PMC6062537; doi:10.1038/s41598-018-29703-0)
Supplement: Supplementary file 1 — Supplementary Images [file 41598_2018_29703_MOESM1_ESM.docx]

**Supplementary data**

**Annexin A2 overexpression associates with colorectal cancer invasiveness and transforming growth factor-ß induced epithelial mesenchymal transition via Src/ANXA2/STAT3**

M Ramos Rocha^1*^, P Barcellos-de-Souza^1^, A C Moraes Sousa-Squiavinato^1^, P Valverde Fernandes^2^, I Martins de Oliveira^2^, M Boroni^3^, J A Morgado-Diaz^1*^

*^1^Cellular and Molecular Oncobiology Program, Instituto Nacional de Câncer, INCA, Rua André Cavalcanti, 37, 20231-050, Rio de Janeiro, Brazil; ^2^Pathology Division - DIPAT, Instituto Nacional de Câncer, INCA, Av Cordeiro da Graça, 156, 20220-400, Rio de Janeiro, Brazil; ^3^Bioinformatics Unit, Instituto Nacional de Câncer, INCA, Rua André Cavalcanti, 37, 20231-050, Rio de Janeiro, Brazil*

***** Corresponding authors. Emails: jmorgado@inca.gov.br (Jose Andres Morgado-Diaz) and rochamr.2@gmail.com (Murilo Ramos Rocha).

**Supplementary Table I.** Association of ANXA2 expression and clinicopathological features


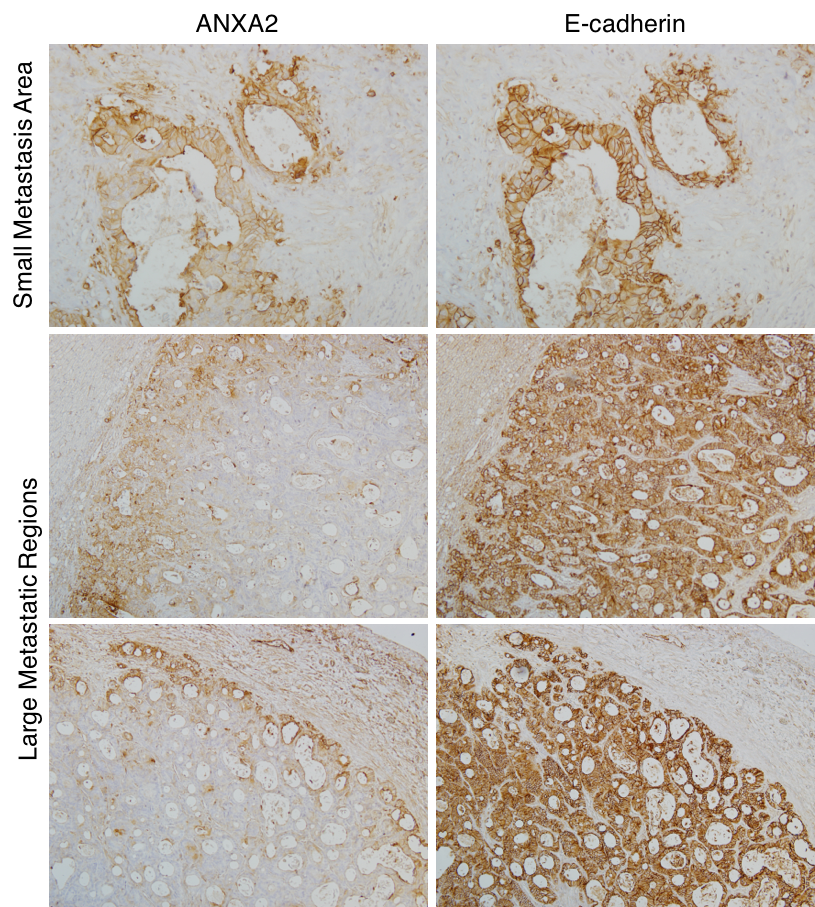


**Supplementary Figure 1.** ANXA2 and E-cadherin staining in liver metastases. Small metastatic area: 60x magnification. Large metastatic regions: 20x magnification.

**
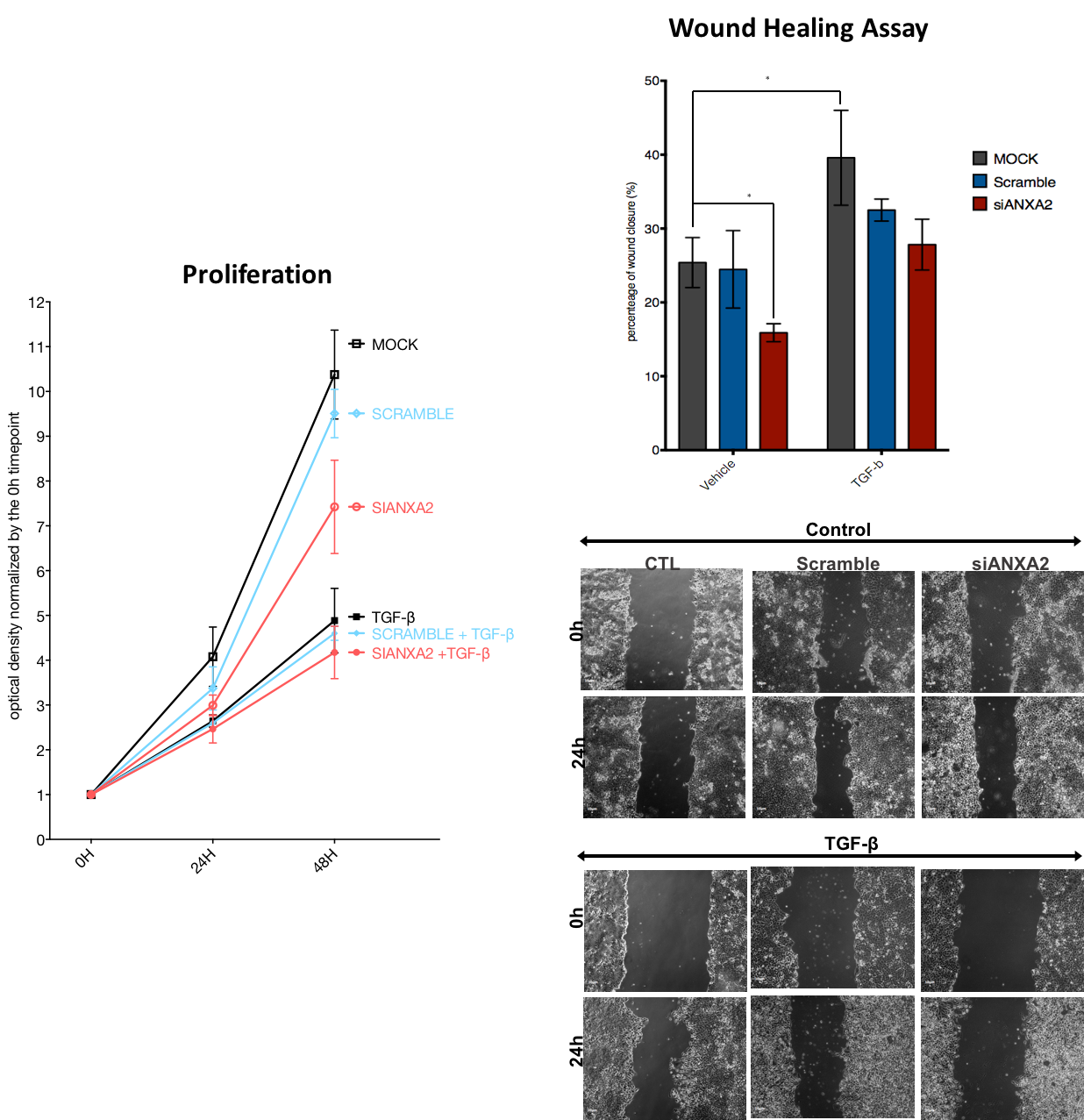
**

**Supplementary Figure 2.** ANXA2 regulates cellular proliferation and migration in a TGF-ß-independent manner. **(A)** ANXA2 silencing hindered cell proliferation 48 h after silencing. TGF-ß, regardless of ANXA2 knockdown, exhibited an anti-proliferative action. **(B)** Wound healing assays indicate that ANXA2 knockdown is associated with reduced wound closure compared to control (no RNA interference). TGF-ß increased wound closure compared to its control (no treatment). * p < 0.05 (paired t-student test).

**
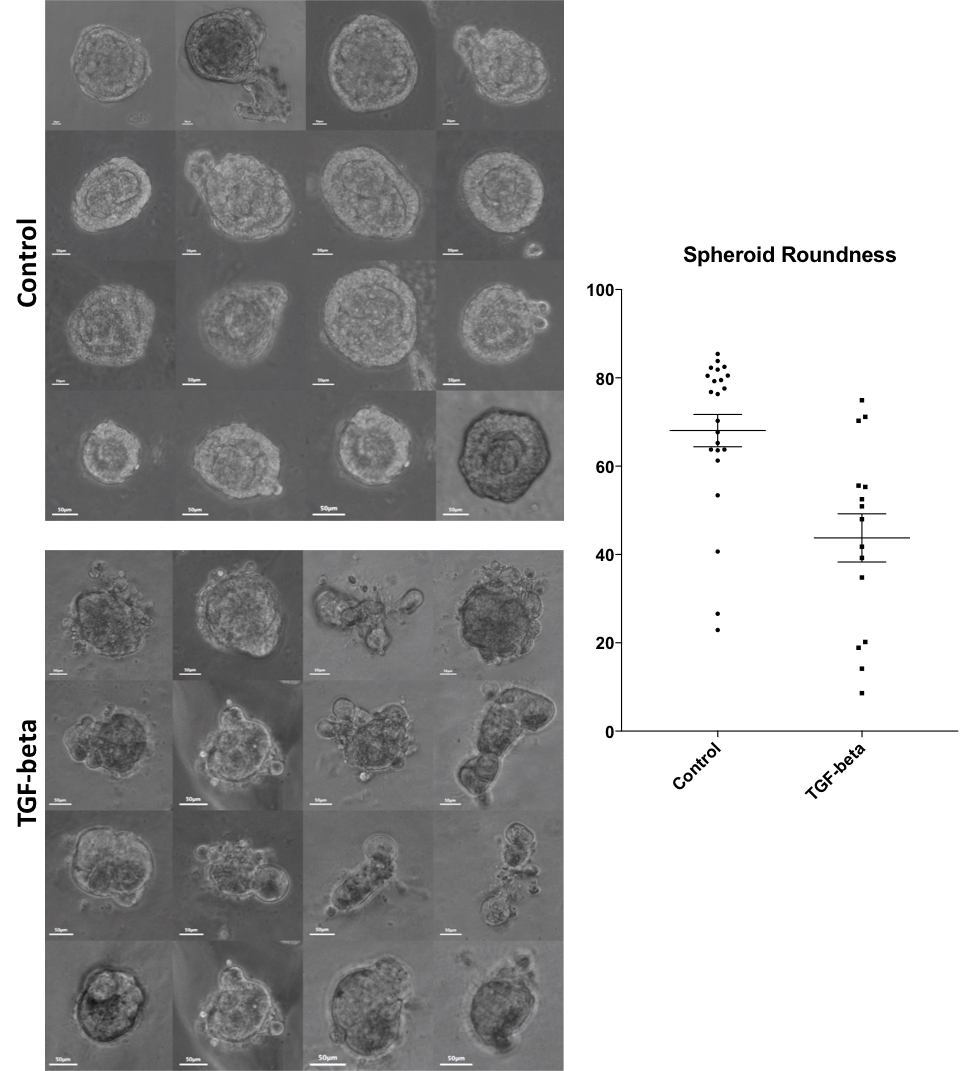
**

**Supplementary Figure 3.** Effect of TGF-ß in a 3D culture system. DIC images of spheroids after 6 days of culture in Matrigel^®^. Spheroid roundness was quantified using the ICY Bioimage Analysis software (Institut Pasteur, Paris, France).
